# Leber Hereditary Optic Neuropathy Caused by the Rare MT-ND1 m.3394T>C Mutation: A Case With Favorable Visual Prognosis and a Literature Review

**DOI:** 10.7759/cureus.103261

**Published:** 2026-02-09

**Authors:** Paulina Mikulenaite, Alvita Vilkeviciute, Almina Stramkauskaite, Ieva Povilaityte, Neringa Jurkute, Rasa Liutkeviciene

**Affiliations:** 1 Department of Ophthalmology, Medical Academy, Lithuanian University of Health Sciences, Kaunas, LTU; 2 Laboratory of Ophthalmology, Neuroscience Institute, Medical Academy, Lithuanian University of Health Sciences, Kaunas, LTU; 3 Biomedical Research Centre, National Institute for Health and Care Research (NIHR), London, GBR; 4 Institute of Ophthalmology, University College London, London, GBR; 5 Department of Ophthalmology, Moorfields Eye Hospital, London, GBR

**Keywords:** aetiology, diagnosis, leber hereditary optic neuropathy, mt-nd1 gene (m.3394t>c), treatment

## Abstract

Leber hereditary optic neuropathy (LHON) is an inherited mitochondrial optic neuropathy characterized by acute or subacute painless central visual loss. Most cases are associated with three primary mitochondrial DNA mutations; however, rare variants remain incompletely characterized. Early diagnosis is essential for appropriate management and genetic counseling. We report the case of a 51-year-old Lithuanian woman who presented with painless, progressive central visual loss. Initial neurological and ophthalmological investigations were unremarkable, and corticosteroid therapy was ineffective. Genetic testing revealed a rare homoplasmic m.3394T>C mutation in the *MT-ND1* gene. The patient was subsequently treated with idebenone and followed for six years. Following initiation of idebenone therapy, the patient demonstrated gradual and sustained improvement in best-corrected visual acuity, reaching 1.0 in both eyes. Visual fields stabilized, and long-term follow-up showed preserved visual function. Optical coherence tomography revealed persistent but stable structural changes, including retinal nerve fiber layer and ganglion cell layer thinning in the affected eye. This case highlights the potential for favorable long-term visual outcomes in patients with LHON associated with rare mitochondrial variants. It underscores the importance of considering hereditary optic neuropathy in patients with painless visual loss and poor response to corticosteroids. Further studies are needed to clarify genotype-phenotype correlations and treatment responsiveness in rare LHON-associated mutations.

## Introduction

Leber's hereditary optic neuropathy (LHON) is a mitochondrial genetic disorder characterized by acute or subacute atrophy of the optic nerves, leading to painless central or centrocecal visual loss [1,2]. It is typically a bilateral disease, and most patients initially present with visual loss in one eye, followed by involvement of the fellow eye most commonly within two to four months [3]. In approximately 20% of cases, both eyes are affected simultaneously [4]. LHON preferentially affects young adults, with a peak age of onset in the second and third decades of life [5]. Visual deterioration is often severe, with visual acuity (VA) typically worse than 20/200 in most patients, and approximately 90% of affected individuals experience significant vision loss before the age of 50 [4,6,7].

LHON is the most common hereditary optic nerve disease, with an estimated prevalence ranging from 1:45,000 to 1:65,000 in Europe [8,9]. Among mutation carriers, males are approximately five times more likely to be affected than females, which is thought to be related to additional genetic and environmental modifiers influencing phenotypic expression [5].

LHON is caused by pathogenic variants in mitochondrial DNA (mtDNA) and is transmitted exclusively through maternal inheritance [5,10]. More than 90% of cases are associated with one of three primary point mutations: m.3460G>A (*MT-ND1*), m.11778G>A (*MT-ND4*), and m.14484T>C (*MT-ND6*) [11-13]. However, these primary mutations demonstrate incomplete penetrance, and the presence of a pathogenic variant alone is often insufficient to cause clinical disease [10,14]. Accumulating evidence suggests that nuclear genetic background, additional mitochondrial variants, and environmental factors together modulate disease expression [10,14,15].

One of the reported mitochondrial genetic modifiers is the m.3394T>C mutation, which has been shown to enhance the phenotypic expression of the primary m.11778G>A mutation and contribute to LHON progression [10,14,16-18]. Higher disease penetrance has been observed in individuals carrying both m.3394T>C and m.11778G>A mutations compared with those harboring the m.3394T>C mutation alone [19]. Accordingly, a strong association between these two variants has been described in the literature [16,20]. Nevertheless, the m.3394T>C mutation has also been reported as potentially deleterious on its own [16].

The m.3394T>C mutation is considered rare, with a reported prevalence of up to 3% in a cohort of nearly 2,000 Chinese LHON probands and approximately 2% in a cohort of 58 Caucasian probands [19,21]. This variant results in the substitution of tyrosine with histidine at position 30 (Y30H) in the MT-ND1 protein, a key subunit of mitochondrial respiratory chain complex I [19]. The Y30 residue interacts with NDUFA1 (NADH dehydrogenase (ubiquinone) 1 alpha subcomplex subunit 1), and this interaction is disrupted by the m.3394T>C mutation, leading to structural and functional alterations of complex I. Experimental data indicate that this mutation is associated with reduced ATP production, decreased mitochondrial membrane potential, and increased generation of reactive oxygen species (ROS) [19].

Because of the rarity of this variant and the limited number of detailed clinical reports, data on the long-term clinical course and treatment response in patients carrying the m.3394T>C mutation remain scarce. To the best of our knowledge, we have identified only one patient with this mutation in our cohort. Therefore, in this article, we present a detailed case report of a patient diagnosed with the rare mtDNA point mutation m.3394T>C, previously described in the literature [16-18,20,21], with long-term follow-up and a favorable visual outcome after idebenone therapy, together with a brief review of relevant LHON literature.

## Case presentation

We report the case of a 51-year-old Lithuanian woman who presented to the Emergency Department in April 2019 with a complaint of decreased VA in her right eye, described as “seeing through fog.” Her ocular history was notable for high myopia (-6.00 D in both eyes), corrected with spectacles.

Initial presentation (week 0)

At presentation, best-corrected VA (BCVA) was 0.6 in the right eye and 0.9 in the left eye. Intraocular pressure was within normal limits bilaterally. Slit-lamp examination revealed no abnormalities in the anterior segments of either eye. Fundoscopic examination showed minimally blurred margins of the right optic nerve head, while the left optic nerve head demonstrated a myopic conus. The macula and peripheral retina appeared normal in both eyes.

A neurological consultation was obtained. Brain computed tomography (CT) showed no pathological changes, and clinical assessment revealed no signs suggestive of optic neuritis. Based on these findings, a provisional diagnosis of “glaucoma suspect” in the right eye was made.

Early disease progression (week 1)

One week later, the patient returned reporting further deterioration in visual acuity and reduced colour contrast sensitivity in the right eye. BCVA in the right eye had declined to 0.01. Visual acuity in the left eye was 0.04, improving to 1.0 with a correction of -6.50 D.

**Figure 1:**
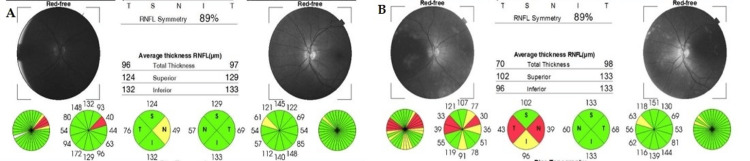
Optical Coherence Tomography of the Peripapillary Retinal Nerve Fiber Layer in Both Eyes. (a) One week after symptom onset. Right eye: superior quadrant 124 μm, nasal 49 μm, inferior 132 μm, and temporal 76 μm. Left eye: superior quadrant 129 μm, nasal 57 μm, inferior 133 μm, and temporal 69 μm. (b) Fifteen months after symptom onset. Right eye: nasal quadrant 39 μm, inferior 96 μm, temporal 43 μm, and superior 102 μm. Atrophy of the nasal and temporal quadrants and subatrophy of the inferior quadrant are observed. Left eye: nasal quadrant 60 μm, inferior 133 μm, temporal 68 μm, and superior 133 μm. The image was generated using the built-in IMAGEnet 6 software (Topcon Corporation, Tokyo, Japan) of the Topcon DRI OCT Triton system.

Optical CT (OCT) revealed nasal quadrant retinal nerve fibre layer (RNFL) atrophy in the right eye, with no abnormalities in the left eye (Figure 1a). Macular OCT was unremarkable. Visual field testing demonstrated a central scotoma in the right eye.

Diagnostic evaluation (weeks 2-4)

Further neurological and otoneurological evaluations revealed no abnormalities. Magnetic resonance imaging (MRI) excluded demyelinating disease. Visual evoked potentials (VEP) demonstrated mild deformation of both P100 waves with marginal latency abnormalities.

Based on progressive painless visual loss, impaired colour perception, central scotoma, abnormal VEP findings, and optic nerve changes, a working diagnosis of optic neuritis was established.

Initial treatment (month 1)

The patient received intravenous methylprednisolone (1 g/day for three days), followed by oral prednisolone tapering. No improvement in visual acuity was observed.

Genetic diagnosis and targeted therapy (month 2)

Given the lack of response to corticosteroids and progressive bilateral involvement, hereditary optic neuropathy was suspected. Mitochondrial genome sequencing revealed a homoplasmic pathogenic variant m.3394T>C, p.(Ile187Thr), in the *MT-ND1* gene.

Treatment with idebenone (900 mg/day) was initiated.

Follow-up and visual recovery

After six months, BCVA improved to 0.6 in the right eye and 0.8 in the left eye. At 13 months, BCVA in the right eye reached 0.8. After 15 months, BCVA in both eyes reached 1.0.

**Figure 2:**
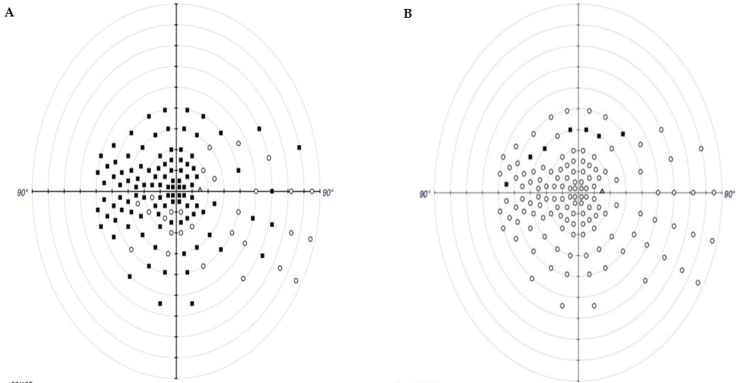
Visual Field Testing of the Right Eye. (a) One week after symptom onset. Central scotoma is present, consistent with acute optic nerve dysfunction. (b) Fifteen months after symptom onset. Partial resolution of the central scotoma with residual nonspecific visual field defects, indicating functional stabilization. The image was generated using the Zeiss Humphrey Field Analyzer 3 (HFA 3) software (Carl Zeiss Meditec, Jena, Germany).

OCT showed persistent RNFL atrophy in the right eye (Figure 1b), while macular OCT remained normal. Visual field testing revealed nonspecific scotomas (Figure 2).

**Figure 3:**
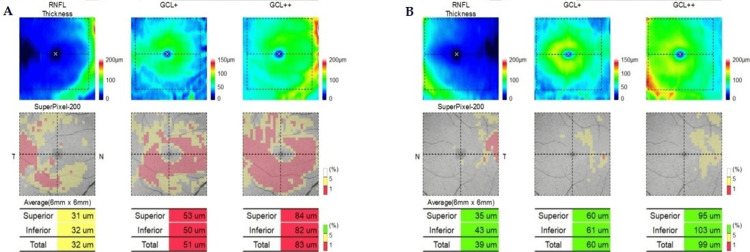
Optical Coherence Tomography of the Ganglion Cell Layer at 13 Months After Symptom Onset. (a) Right eye. Slightly decreased and asymmetrical peripapillary retinal nerve fiber layer (RNFL) thickness: superior 31 μm and inferior 32 μm. Thinning of ganglion cell layers (GCL+ and GCL++) was found in both superior and inferior parts. The total count of GCL+ was 51 μm, and GCL++ was 83 μm. (b) Left eye. Normal thickness of RNFL in both superior and inferior parts. Normal values of total GCL+ (60 μm) and GCL++ (99 μm). The image was generated using the built-in IMAGEnet 6 software (Topcon Corporation, Tokyo, Japan) of the Topcon DRI OCT Triton system.

Ganglion cell layer imaging demonstrated mild thinning in the right eye (Figure 3).

Long-term outcome

**Figure 4:**
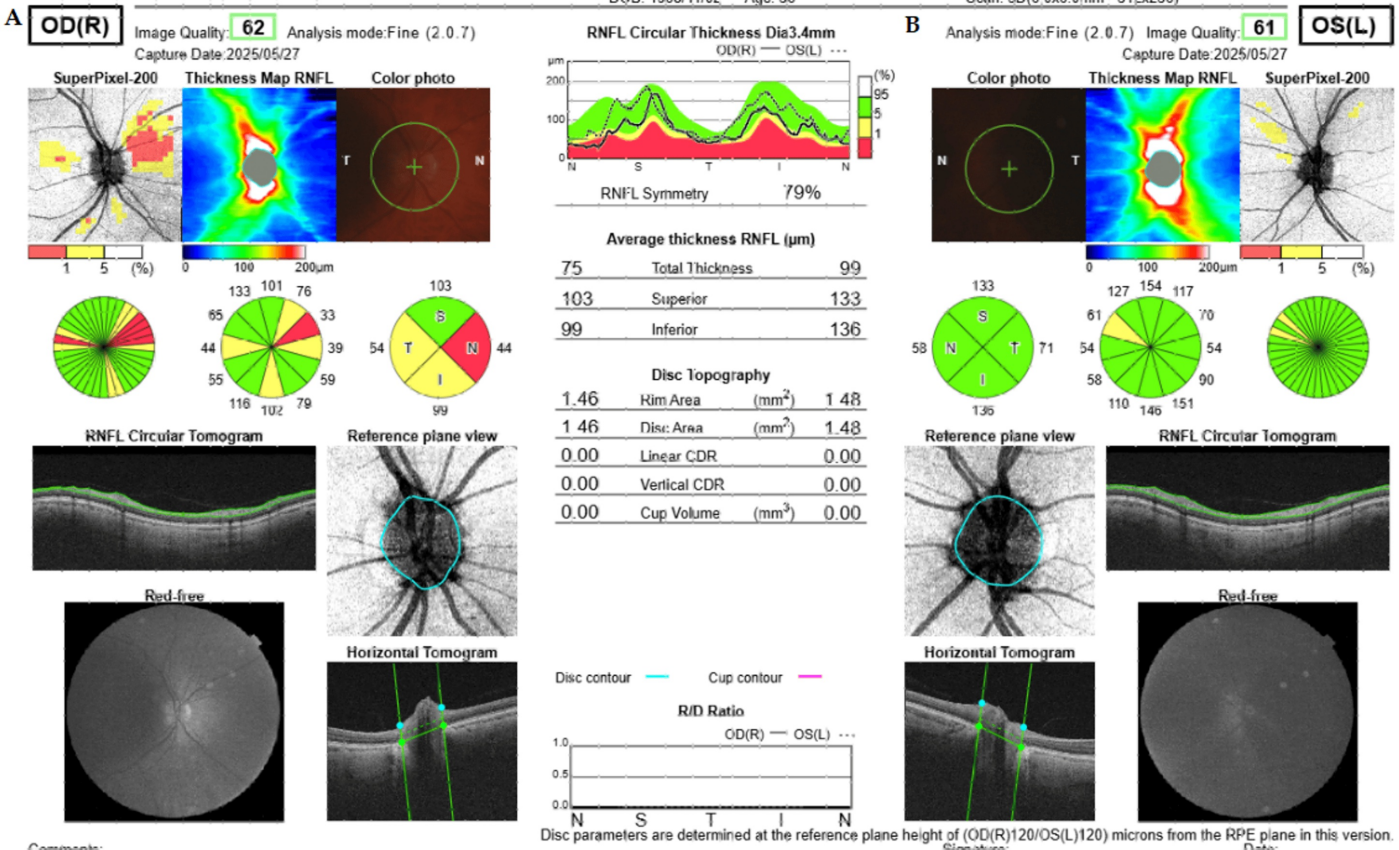
Optical Coherence Tomography of the Peripapillary Retinal Nerve Fiber Layer in Both Eyes Six Years After Symptom Onset. (a) Right eye. Nasal quadrant 44 μm, inferior 99 μm, temporal 54 μm, and superior 103 μm. Atrophy of the nasal quadrant and subatrophy of the temporal and inferior quadrants are observed. (b) Left eye. Nasal quadrant 58 μm, inferior 136 μm, temporal 71 μm, and superior 133 μm. The image was generated using the built-in IMAGEnet 6 software (Topcon Corporation, Tokyo, Japan) of the Topcon DRI OCT Triton system.

Idebenone therapy was continued for 24 months and discontinued after sustained visual stabilization. At the most recent follow-up - six years after onset and more than three years after treatment cessation - BCVA remains stable at 1.0 in both eyes, with no evidence of disease progression on OCT or visual field testing (Figure 4).

A summary of the patient's clinical course, diagnostic evaluations, treatment, and outcomes is provided in Table 1.

**Table 1:** Summary of Clinical Course, Investigations, and Treatment. BCVA – best-corrected visual acuity; OCT – optical coherence tomography; RNFL – retinal nerve fiber layer; GCL – ganglion cell layer; VF – visual field; VEP – visual evoked potentials; LHON – Leber hereditary optic neuropathy. Time points are referenced from symptom onset. Visual acuity values are reported as decimal equivalents. Structural changes were assessed using OCT, and functional outcomes were evaluated using visual field testing and electrophysiological examinations.

Time Point	Clinical Findings	Key Investigations	Treatment	Outcome
April 2019 (Week 0)	Painless blurred vision (RE)	BCVA: RE 0.6, LE 0.9; Normal CT	—	Glaucoma suspect
Week 1	Rapid VA decline, colour vision loss	OCT: RNFL atrophy (RE); VF: central scotoma	—	Progression
Weeks 2–4	Persistent visual loss	Normal MRI; Abnormal VEP	—	Suspected optic neuritis
Month 1	No improvement	—	IV + oral steroids	No response
Month 2	LHON suspected	mtDNA: m.3394T>C	Idebenone	Diagnosis confirmed
Month 6	Partial recovery	BCVA: RE 0.6, LE 0.8	Continued	Improvement
Month 13	Continued recovery	BCVA: RE 0.8	Continued	Stabilization
Month 15	Stable vision	OCT/GCL changes	Continued	BCVA 1.0
Month 24	Stable	Stable OCT/VF	Discontinued	Remission
Year 6	Long-term stability	No progression	None	BCVA 1.0

## Discussion

We report a case of LHON caused by the rare mitochondrial DNA point mutation m.3394T>C. This variant has been reported in approximately 2.7% of a cohort of nearly 2,000 Chinese patients with LHON [19]. Previous studies have demonstrated that both disease penetrance and the degree of mitochondrial dysfunction depend on whether individuals carry the m.3394T>C mutation alone or in combination with primary LHON mutations, such as m.11778G>A or m.14484T>C. Individuals harboring only the m.3394T>C mutation generally exhibit lower penetrance and milder mitochondrial dysfunction compared with those carrying both m.3394T>C and m.11778G>A mutations [19].

It has been suggested that single mitochondrial mutations, including m.3394T>C and m.11778G>A, are often insufficient to produce the full clinical phenotype in isolation. Instead, nuclear and additional mitochondrial genetic modifiers appear to play an important role in determining disease expression [10,22,23]. Genetic evidence further indicates that the m.3394T>C mutation may modify mitochondrial function in a manner that enhances the phenotypic expression of m.11778G>A [19].

In the present case, the patient carried only the m.3394T>C mutation. Experimental data suggest that individuals with this isolated variant may retain relatively higher ATP production capacity compared with those harboring combined mutations, potentially resulting in less pronounced retinal ganglion cell degeneration [19]. This mechanism may partly explain the relatively favorable clinical course observed in our patient.

The m.3394T>C mutation has previously been associated not only with LHON but also with other clinical conditions, including sensorineural hearing loss and metabolic disorders [24,25]. However, no such abnormalities were observed in our patient during long-term follow-up.

Visual recovery in LHON has been reported in some patients and is thought to be related to the preservation or reorganization of small functional retinal areas within central scotomas [26,27]. When residual “islands” of normal vision develop within the affected region, partial or complete recovery of VA may occur. This mechanism may have contributed to the favorable visual outcome in our patient, whose BCVA recovered to 1.0 in both eyes. In contrast, extensive involvement of the central visual field is typically associated with persistent visual impairment [26].

Following idebenone therapy, our patient demonstrated gradual improvement in VA and visual field stabilization. Structural assessment using OCT revealed residual optic nerve changes, including borderline RNFL thickness and reduced ganglion cell layer (GCL) measurements in the right eye. Although partial resolution of subatrophy in the temporal quadrant was observed, these findings indicate that structural damage persisted despite functional recovery. This dissociation between structural and functional outcomes has been described previously in LHON and may reflect compensatory neuroplastic mechanisms or improved mitochondrial function in surviving retinal ganglion cells.

Several limitations should be considered when interpreting these findings. This report describes a single patient, and therefore causal relationships between genotype, treatment, and clinical outcome cannot be established. In addition, the observed response to idebenone cannot be generalized to all patients carrying the m.3394T>C mutation.

## Conclusions

In conclusion, we present a case of LHON associated with the rare m.3394T>C (p.Ile187Thr) mutation in the *MT-ND1 *gene, characterized by long-term visual recovery and clinical stability following idebenone therapy. This case highlights the potential variability in disease expression and prognosis associated with rare mitochondrial variants. Further studies involving larger cohorts and longer follow-up are required to better clarify the clinical significance and therapeutic responsiveness of this mutation.
